# Preliminary Insights into the Non-Volatile Constituents of *Commiphora ornifolia* (Balf.f.) J.B.Gillett Oleogum Resin from Socotra Island

**DOI:** 10.3390/plants14192999

**Published:** 2025-09-28

**Authors:** Martina Bortolami, Dario La Montagna, Chiara Toniolo, Fabio Sciubba, Adriano Patriarca, Tiziana Moretti, Ilaria Serafini, Francesco Mura, Emma Cocco, Petr Maděra, Kay Van Damme, Stefania Garzoli, Luca Santi, Fabio Attorre, Daniela De Vita

**Affiliations:** 1 Department of Environmental Biology, Sapienza University of Rome, Piazzale Aldo Moro 5, 00185 Rome, Italy; martina.bortolami@uniroma1.it (M.B.); dario.lamontagna@uniroma1.it (D.L.M.); chiara.toniolo@uniroma1.it (C.T.); fabio.sciubba@uniroma1.it (F.S.); moretti.1056831@studenti.uniroma1.it (T.M.); ilaria.serafini@uniroma1.it (I.S.); f.mura@uniroma1.it (F.M.); l.santi@uniroma1.it (L.S.); fabio.attorre@uniroma1.it (F.A.); 2 NMR-Based Metabolomics Laboratory (NMLab), Sapienza University of Rome, Piazzale Aldo Moro 5, 00185 Rome, Italy; adriano.patriarca@uniroma1.it; 3 Interdepartmental Center of Applied Sciences for the Protection of the Environment and Cultural Heritage (CIABC), Sapienza University of Rome, Piazzale Aldo Moro 5, 00185 Rome, Italy; 4 Department of Chemistry, Sapienza University of Rome, Piazzale Aldo Moro 5, 00185 Rome, Italy; 5 Laboratory of Economic and Pharmaceutical Botany, Department of Life and Environmental Sciences, University of Cagliari, V.le S. Ignazio da Laconi 13, 09123 Cagliari, Italy; emma.cocco@unica.it; 6 Department of Forest Botany, Dendrology and Geobiocoenology (FFWT), Mendel University, Zemedelska 1, 61300 Brno, Czech Republic; petr.madera@mendelu.cz (P.M.); kay.vandamme@gmail.com (K.V.D.); 7 Centre for Academic Heritage and Archives & Ghent University Botanical Garden, Ghent University, K.L. Ledeganckstraat 35, 9000 Ghent, Belgium; 8 Department of Chemistry and Technologies of Drug, Sapienza University of Rome, Piazzale Aldo Moro 5, 00185 Rome, Italy; stefania.garzoli@uniroma1.it

**Keywords:** *Commiphora ornifolia* (Balf.f.) J.B.Gillett, oleogum resin, yangambin, lignans, Socotra

## Abstract

Natural resins are complex mixtures of secondary metabolites produced by many plants in response to stress or injury and have long been used for their antimicrobial, anti-inflammatory, and antioxidant properties. Among resin-producing genera, *Commiphora* Jacq. (Burseraceae) stands out for the traditional and medicinal relevance of its oleogum resins, commonly known as myrrh. In this study, we investigated, for the first time, the non-volatile fraction of the oleogum resin of *Commiphora ornifolia* (Balf.f.) J.B.Gillett, which is an endemic species of Socotra Island. Ethanol extraction followed by chromatographic and spectroscopic analysis (HPLC-DAD, NMR, HRMS) led to the isolation of (+)-yangambin, a furofuran lignan not previously reported in this species. Quantitative analysis showed yangambin to be present in all eight resin samples analyzed, at concentrations ranging from 3.50 (±0.02) to 9.05% (±0.19) of the ethanol extract. In addition, the analysis of the hydrolyzed polysaccharide fraction revealed the presence of arabinose, rhamnose, galactose, and galacturonic acid. These preliminary findings highlight the phytochemical richness of *C. ornifolia* oleogum resin and suggest the presence of other potentially bioactive compounds. The presence of yangambin, known for various pharmacological activities, supports further phytochemical and biological studies on this largely unexplored species.

## 1. Introduction

Natural resins are complex mixtures of lipophilic plant exudates produced by specialized secretory tissues, often in response to wounding or environmental stress. Chemically, they are composed of a heterogeneous array of low-molecular-weight molecules, such as terpenoids, steroids, and lignans, which may be associated with volatile oils and gums depending on their botanical origin and physiological function [1,2,3]. These metabolites not only contribute to the plant’s defense mechanisms but have also attracted considerable pharmacological interest due to their antimicrobial, anti-inflammatory, antioxidant, and anticancer activities [3,4,5]. In traditional medicine, resins have been extensively used as wound-healing agents, analgesics, and antiseptics.

Among resin-producing genera, *Commiphora* Jacq. holds a particularly prominent place due to the medicinal and aromatic properties of its oleogum resins, commonly referred to as myrrh. These resins are exuded from stem incisions and consist of a water-soluble portion (30–60%), an essential oil fraction (3–8%), and an alcohol-soluble resin (25–40%) rich in terpenoids and steroids [6].

The genus *Commiphora*, belonging to the Burseraceae family, comprises over 200 species of shrubs and small trees, distributed mainly in arid and semi-arid regions of Africa, Arabia, the Indian subcontinent, and Southern America [7], but a remarkable diversity of endemism is found on the island of Socotra, a UNESCO World Heritage Site known for its exceptional botanical biodiversity [8]. In fact, on the island of Socotra, five species of the genus *Commiphora* grow: *Commiphora kua* (R.Br. ex Royle) Vollesen, *C. ornifolia* (Balf.f.) J.B.Gillett, *C. planifrons* Engl., *C. parvifolia* Engl., and *C. socotrana* Engl. Of these, only *C. kua* is non-endemic, as it is also found in the Arabian Peninsula [9].

**Figure 1:**
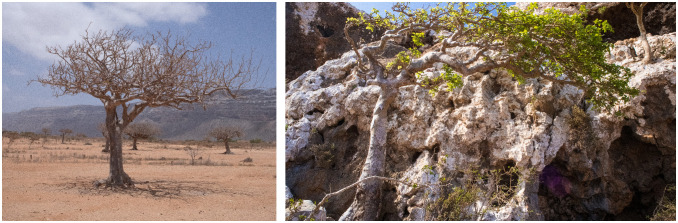
*Commiphora ornifolia* (Balf.f.) J.B.Gillett is one of the endemic myrrh trees of Socotra Island (Yemen). Photos by D.L.M.

In particular, *C. ornifolia* is a tree that grows particularly on the limestone escarpments up to 1050 m above sea level. The plant has smooth grey bark and branches that are rather tortuous. The leaves are odd-pinnate with three to seven leaflets (4–9 × 2–6 cm), which are ovate or slightly elliptic and glabrous to thinly hairy or velvety. The small flowers (c. 5 mm) are arranged in raceme-like cymes, and the fruits are fleshy, globose, and bright green, resembling small grapes (Figure 1) [10].

*C. ornifolia* is highly valued by the local population of Socotra, who uses its bark and the aromatic resin for traditional medicinal purposes [10,11]. Particularly, the bark and inner bark are exploited to produce the most commonly used powder dressing, which is applied to treat sores, ulcers, and all types of wounds in both humans and livestock; moreover, it is used as an antiseptic, for treating diarrhea and dysentery, and as an emmenagogue [11]. The sap is used as a remedy for different types of skin ailments and sores; the resin is inserted into the tooth cavity as a remedy for toothache [10].

Despite the interest in this species, previous phytochemical studies have exclusively addressed the volatile components of the oleogum resin of *C. ornifolia* [12,13]. In addition, in our previous work [14] we investigated its volatile fraction via SPME-GC/MS.

To the best of our knowledge, the non-volatile fraction of the oleogum resin of *C. ornifolia* has never been investigated, and this prompted us to undertake a study focused on this component. In the present work, we concentrated on the analysis of both the non-volatile apolar and polar components.

## 2. Results and Discussion

### 2.1. Extraction of Secondary Metabolites and Isolation of Yangambin

**Figure 2:**
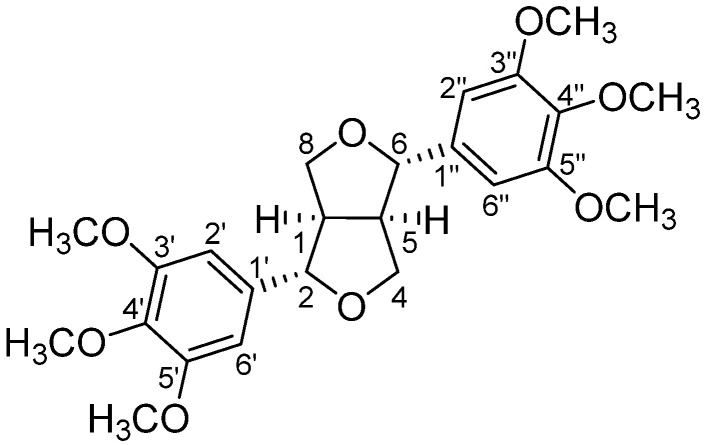
(+)-yangambin (**1**) structure.

For the phytochemical study, the non-volatile fraction of different samples of the oleogum resin of *C. ornifolia*, collected on Socotra Island (Figure S1), was macerated in ethanol. This kind of extraction, at room temperature, should avoid the degradation of thermolabile compounds eventually present in the resin [15]. The maceration with ethanol allowed us to obtain an extract with a complex composition, as suggested by preliminary high-performance thin-layer chromatography (HTLC) and high-performance liquid chromatography (HPLC) analyses. The extract from the oleogum resin sample collected at Killesan (RC7, Figure S1) was then subjected to column chromatography. The subsequent separation by preparative liquid chromatography (PLC) led to the isolation of the compound (+)-yangambin (**1**) (Figure 2), a furofuran lignan reported for the first time in this species.

Its structure was confirmed by ^1^H and ^13^C NMR and HRMS analyses. In the ^1^H NMR spectrum, the chemical shifts and the coupling constants (*J*) of the signals related to the furofuran moiety allowed us to unambiguously confirm the structure of yangambin [16], excluding the structure of epiyangambin [16] or diayangambin [17] for the isolated compound. In particular, in yangambin H-2 and H-6 are equivalent, leading to a doublet at 4.75 ppm, with a *J* of 4.2 Hz, differently to epiyangambin, in which H-2 and H-6, respectively, a doublet at 4.40 ppm, with a *J* of 7.0 Hz, and a doublet at 4.81 ppm, with a *J* of 5.3 Hz. Otherwise, in diayangambin, even if H-2 and H-6 are equivalent, their signal appears downfield at 4.92 ppm, while the doublet of doublets related to H-4_ax_/H-8_ax_ and H-4_eq_/H-8_eq_ are both upfield (i.e., 3.74 and 3.59 ppm in diayangambin vs. 4.31 and 3.94 ppm in yangambin). Even the signals in the ^13^C NMR spectrum are consistent with those reported in the literature for yangambin. In this case, as expected based on the chemical structure, C-2 and C-6, as well as C-4 and C-8, and C-1 and C-5 are equivalent (differently to the same carbons in epiyangambin), giving signals at 86.1, 72.1, and 54.6 ppm, respectively (different position compared to the same carbons in diayangambin).

After the isolation of yangambin from the oleogum sample collected at Killesan (Figure S1), an HPLC-DAD titration curve was obtained and used to quantify this furofuran lignan in the ethanol extracts of all the oleogum resin samples of *C. ornifolia*, collected at different times and areas on Socotra Island (Figure S1). Table 1 reported the obtained results expressed in *w*/*w* percentages of yangambin in the total ethanol extracts. Interestingly, yangambin was found in all the analyzed samples, in variable amounts, ranging from 3.50 (±0.02) to 9.05% (±0.19).

To the best of our knowledge, yangambin has previously been reported in the genus *Commiphora* only from the species *C. wightii* (Arn.) Bhandari [18]. Moreover, its diastereoisomer diayangambin was found in the oleogum resin of *C. wightii* [19,20] and tentatively identified in its bark [21]. Two works in the literature suggested the presence of diayangambin in the resins of *C. myrrha* (T.Nees) Engl. [22,23]; however, in both cases the compound was not isolated, and it was tentatively identified only based on mass spectrometry data. Other furofuran lignans, e.g., epi-syringaresinol, epi-magnolin, and diasesartemin, were reported in *C. wightii* [18,20,24,25] and *C. myrrha* [26].

More broadly, our results represent the first evidence of lignans in *C. ornifolia* species. This finding underscores the ecological and biochemical significance of lignans in plants, prompting a broader contextualization of their occurrence and roles. Lignans are a large and heterogeneous class of secondary metabolites widely diffused in the plant kingdom and belonging to the group of phenols [27] formed by the dimerization of two phenylpropanoid units and classified in eight groups based on their chemical structure (carbon skeleton, oxygen incorporation, and cyclization pattern) [28]. Furofuran lignans, characterized by a 2,6-diaryl-3,7-dioxabicyclo [3.3.0] octane skeleton, constitute a major subclass within the lignan family of natural products. They are also referred to as bisepoxylignans, linked through the 8–8′, 7-O-9′, or 9-O-7′ positions [29]. Furofuran lignans have been reported in 53 species belonging to 41 genera and 27 different plant families, such as Lamiaceae and Lauraceae [30]. They occur in various parts of the plants, including roots, stems, leaves, bulbs, bark, and seeds [29]. In regard to their biological function in plants, some authors have proposed a defense action, in the light of their insecticidal and antiviral activities [31,32]. However, the current data supporting this hypothesis are still limited, and therefore further studies on the biological roles of lignans in plants are needed.

On the other hand, the pharmacological properties of a large number of natural lignans are the subject of numerous studies, both in vitro and in vivo, and, to a lesser extent, in human clinical trials. Indeed, many lignans show antiviral [33,34], anticancer [35,36,37], anti-inflammatory [38], and antioxidant activities, along with anti-neurodegenerative [39], anti-diabetic [40], and anti-obesity properties [41], through different and sometimes interconnected mechanisms, justifying their protective role against chronic diseases.

More specifically, furofuran lignans have been shown to exhibit a wide range of significant biological activities, including antioxidant, anti-inflammatory, cytotoxic, and antimicrobial actions [29]. Looking more closely at yangambin, it is an interesting compound with relevant pharmacological properties. It shows cardiovascular activities, being a platelet-activating factor (PAF) receptor antagonist and preventing cardiovascular collapse during anaphylactic and endotoxic or septic shocks [42,43]. Furthermore, it presents additional anti-allergic properties [44], antitumor effects [45,46], antileishmanial activity [47,48,49], and depressant effects on the central nervous system [50].

Although preliminary, our findings highlight the need for a more comprehensive investigation of the secondary metabolite profile of *C. ornifolia* resin. The presence of yangambin and its known bioactivities support the relevance of pursuing further phytochemical studies, as well as research on the ecological functions in the plant and the potential pharmacological applications of this and other lignans.

### 2.2. Extraction and Analyses of Monosaccharides

Some evidence related to the pharmaceutical activities of the polysaccharide fractions in *Commiphora* species, such as the inhibitory activity against α-D-glucosidase, the ability to enhance the phagocytosys of polymorphonuclear leukocytes [51], and the osteoclastogenesis inhibition [52], underline the importance of clarifying the monosaccharide composition of *C. ornifolia*, which to date has never been studied.

The monosaccharide composition of a polysaccharide is typically determined through acid hydrolysis, followed by the analysis of the resulting monosaccharides [53]. This approach enables the identification and quantification of the individual sugar units that form the polysaccharide backbone, providing essential insights into its structure and helping to clarify structure–function relationships. The first step involves the cleavage of glycosidic bonds through complete hydrolysis, most commonly achieved using acidic conditions. The most frequently used acids include trifluoroacetic acid (TFA) [54] and hydrochloric acid [55]. TFA, in particular, is preferred for its ease of removal by evaporation and compatibility with analytical systems [56]. Based on literature data and previous experience in the laboratory, TFA was selected for this analysis.

**Figure 3:**
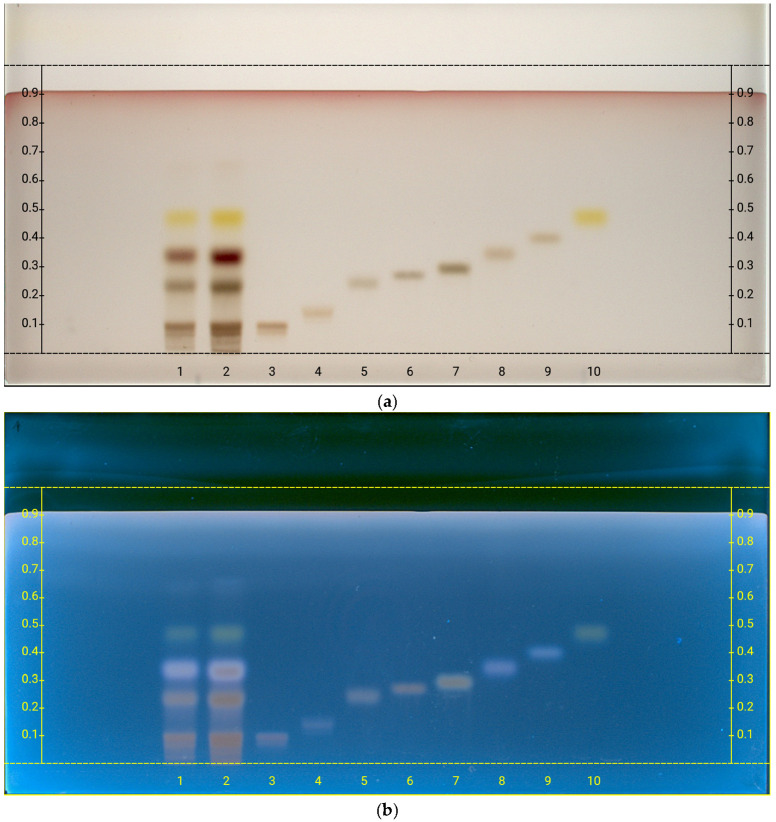
HPTLC fingerprints of monosaccharides produced from TFA hydrolysis of polysaccarides obtained from the oleogum resin, visualized under (**a**) white light and (**b**) UV 366 nm, after derivatization with sulphuric anisaldehyde. Mobile phase: acetonitrile/water (85:15 *v*/*v*). Track assignments: 1 hydrolyzed olegum extract 3.0 μL; 2 hydrolyzed olegum extract 5.0 μL; 3 galacturonic acid; 4 glucuronic acid; 5 galactose; 6 glucose; 7 fructose; 8 arabinose; 9 xylose; 10 rhamnose.

After the hydrolysis of the polysaccarides obtained from the oleogum resin, a targeted analysis was carried out to detect specific monosaccharides. The selected sugars (galacturonic acid, glucuronic acid, galactose, fructose, glucose, arabinose, rhamnose, and xylose) were chosen based on literature [5,51,52,57,58] data to reflect the expected monomeric units. For this purpose, after the hydrolysis of polysaccharides by TFA, high-performance thin-layer chromatography (HPTLC) has been performed for the analysis of monosaccharides [54,59]. The results of the analysis indicated that the following sugars are present (Figure 3): galacturonic acid (R*_f_* = 0.100), galactose (R*_f_* = 0.242), arabinose (R*_f_* = 0.351), and rhamnose (R*_f_* = 0.473).

These findings were further validated through the ^1^H NMR analysis (Figure S2) of the hydrolyzed product, supporting the results obtained by HPTLC. Carbohydrates were uniquely identified by NMR, based on the chemical shift, multiplicity, and *J*-coupling constants of the one-dimensional proton spectrum (Table 2 and Figure S2), and confirmed by data in the literature and databases [60,61,62].

As reported in Table 3, arabinose is the main monosaccharide component of the polysaccharides, followed by rhamnose, galactose, and galacturonic acid. Signals related to mannose at 5.17 and 4.9 ppm were found to be under the limit of detection. All the identified sugars are consistent with those previously reported in oleogum resins from other *Commiphora* species. [51,52,57,58]. Based on literature data, the oleogum of different *Commiphora* species generally contains arabinose and galactose as the two major monosaccharides, but their relative ratio as well as the presence and amount of other monosaccharides are variable [58]. Dahi et al. characterized the water-soluble fraction of *C. africana* and reported arabinose (47%), galactose (28%), and galacturonic acid (7%) as major monosaccharides, together with a minor amount of mannose, xylose, glucosamine, and fucose (about 4–5% each) [57]. Boual et al. described *C. myrrha*’s monosaccharide composition, formed by galactose (45%), arabinose (44%), xylose (6%), and mannose (5%) [51]. These data are only partially in agreement with those found by Hwang and colleagues for *C. myrrha* polysaccharides, mainly composed of galactose (65.6 mol%), arabinose (29.8 mol%), glucoronic acid (2.86 mol%), rhamnose (0.96 mol%), and fucose (0.78 mol%) [52], or by Hough et al., who identified D-galactose, L-arabinose, and 4-methyl D-glucuronic acid (in relative proportions 4:1:3) in *C. myrrha* hydrolyzed polysaccharides [63]. These data suggest a variability in the polysaccharide composition not only in different *Commiphora* species but also in the same species with different geographical origins. In our study, in the hydrolyzed polysaccharide fraction of *C. ornifolia*, besides arabinose and galactose, a relatively high amount of rhamnose was found, which is not generally observed or is present only in low amounts in other *Commiphora* species.

## 3. Materials and Methods

### 3.1. Plant Material

The oleogum resins were collected on Socotra Island in the period of November 2022–April 2023, with GPS-recorded coordinates and altitudes (Figure S1). Botanical identification was carried out by Dr. Dario La Montagna based on morphological comparison with literature data [10]. The sample was collected in glass vials by gently tapping the trees with a knife. The vials were immediately sealed with Teflon-lined caps. Collection and export were authorized by the Environment Protection Authority (EPA) of the Republic of Yemen, which oversees the Socotra Archipelago.

### 3.2. Solvents, Reference Compounds, and Chromatographic Materials

All the solvents and standards used were purchased from Sigma Aldrich and used without further purification. Deuterated solvents (CDCl_3_ and D_2_O) were used for the identification of compounds by NMR Spectroscopy.

For purification by column chromatography silica gel (Merck 40–63 μm particle size) was used as stationary phase. For PLC, silica gel 60 glass plates (Supelco, Belfonte, PA, USA) with L × W 20 cm × 20 cm and fluorescent indicator and a mixture of toluene–acetone (9:0.8 *v*/*v*) were used as a chromatographic system. HPTLC Silica Gel 60 F254 20 × 10 cm plates were purchased from Merck (Darmstadt, Germany).

### 3.3. Instruments and Analytical Methods

NMR analyses were recorded on a Bruker Avance III 400 MHz instrument (Billerica, MA, USA) operating at 9.4 T at 298 K, with the chemical shifts expressed in ppm.

HPLC-DAD analyses were performed with a Nexera XR (Shimadzu, Kyoto, Japan) instrument equipped with photodiode array detector (DAD) SPD-M40 model, an autosampler SIL-40C XR, a quaternary pump (LC-40D XR), a degassing unit (DGU-405), a control system CBM-40, a thermostatically controlled oven (CTO-40S), and a chromatographic column Shim-pack Velox C18 (150 mm × 3.00 mm, 2.7 μm). Milli-Q water (A) and methanol (B) were used as mobile phase, with a flow rate of 0.50 mL/min and a gradient profile as follows: 0 min, 10% B; 0–5 min, 10% B; 5–42 min, 100% B; 42–52 min, 100% B; 52–53 min, 10% B; 53–73 min, and 10% B. The PDA detector recorded one UV–Vis spectrum per second in the range of 190–400 nm.

LC-HRMS analysis was carried out with a Vanquish Core HPLC system equipped with an automatic sampler. Chromatographic separation of analytes was performed on a Phenomenex Kinetex F5, 2.6 μm column, 2.1 × 100 mm. The mobile phases used were MeOH with 0.1% formic acid (A, organic phase) and H_2_O with 0.1% formic acid (B, aqueous phase). The chromatographic gradient was as follows: initial condition of 30% B phase, then B phase was increased to 55% in 3 min, then to 59% in 6 min, and to 100% in 7 min. It was maintained at 100% for 2 min (9 min), then it decreased from 100% to 30% at 10.5 min and was maintained at this condition to 12 min. The run took a total of 15 min. The flow rate was 0.25 mL/min, and the column temperature was kept constant at 35 °C. Mass spectrometry analysis was performed using an Orbitrap Exploris 240 mass spectrometer (Thermo Fischer Scientific Inc., Monza Italy), equipped with a heated electrospray ionization (H-ESI) source, operating in negative ionization. The source parameters were as follows: H-ESI temperature 300 °C, ion transfer tube temperature 280 °C, nebulization voltage of 3.2 kV (-), nebulizer gas 30 a.u., and auxiliary gas 10 a.u. The resolution was set at 120 K, and the scan range was 100–1200 *m*/*z*.

The specific optical rotation of yangambin was recorded with a Jasco P-2000 polarimeter, with a 589 nm sodium lamp, path length of 10 mm, and cell volume of 1 mL. The final value of specific optical rotation (*c* = 0.36, CHCl_3_, T = 29.8 °C) is given as average ± S.D. of five subsequent measurements.

The HPTLC system (Camag, Muttenz, Switzerland) consisted of an automatic sampler ATS 4 for precise and reproducible sample application, an Automatic Developing Chamber 2 (ADC2) for controlled plate development, a TLC Plate Heater III for uniform drying, and a Chromatogram Immersion Device III for derivatization. Detection was performed with a TLC Visualizer under UV 254 nm, UV 366 nm, and white light. Data acquisition and instrument control were managed through the VisionCats software (Version 3.1). For HPTLC analysis, samples and standards were applied to HPTLC plate by an automated “spray-on” technique under a N_2_ flow at a rate of 20 nL/s, with a volume of 1 μL per application for the reference sugars and 3 or 5 μL of hydrolyzed oleogum sample. The mobile phase used was acetonitrile/water (85:15 *v*/*v*). Plate development was carried out using the selected mobile phase for both development and saturation. After development, the plate was dried for 5 min at 120 °C and visualized at UV wavelengths of 254 nm and 366 nm and white light. The slit size was maintained at 5.00 × 0.20 mm, and the scanning speed was set to 20 mm/s. Subsequently, derivatization was performed by immersing the plate in sulphuric anisaldehyde. Prior to documentation under UV light at 366 nm and white light in reflectance mode, the plates were dried on a heating plate at 120 °C for 5 min.

### 3.4. Extraction and Isolation of the Main Components

The resin of RC7 (6 g) was coarsely ground with a mortar and macerated with 96° ethanol (25 mL) for 48 h. After this period, the suspension was filtered, and the residue was subjected to a second ethanol extraction. The two extracts were combined and evaporated under reduced pressure to obtain an oily dry extract (RC7EE, 13% yield), which was subjected to column chromatography. The remaining solid phase (5.1 g) was used for carbohydrate characterization: an aliquot (0.2 g) of this residue was treated with water (10 mL) at 70 °C for 10 min, hot-filtered, and precipitated with ethanol (20 mL). After 48 h at −18 °C, the suspension was centrifuged and air-dried. The whitish solid obtained was used for carbohydrate characterization, without further purification.

#### 3.4.1. Column Chromatography and Isolation of Yangambin from RC7EE

Five hundred milligrams of RC7EE was purified by column chromatography using silica as the stationary phase and a 3:7 *v/v* mixture of ethyl acetate and *n*-hexane as the mobile phase. Fractions of approximately 10 mL were collected, analyzed by TLC, visualized and derivatized with a methanolic solution of sulfuric acid 10% *v/v*, and combined based on their similarity. Five fractions were obtained and analyzed by ^1^H NMR spectroscopy. The fractions showing the highest degree of purity were further purified by preparative liquid chromatography (PLC), leading to the isolation of (+)-yangambin, subsequently recrystallized in ethyl acetate/*n*-hexane.

(+)-yangambin [16]: white solid; ${\left[\alpha \right]}_{D}^{29.8}$ + 17.94 ± 0.25 (*c* 0.36, CHCl_3_) [64,65]; ^1^H NMR (400 MHz, CDCl_3_): δ = 6.57 (s, 4H, H-2′, H-6′, H-2″, H-6″), 4.75 (d, *J* = 4.2 Hz, 2H, H-2, H-6), 4.31 (dd, *J* = 9.08 Hz, 6.84 Hz, 2H, H-4_eq_, H-8_eq_), 3.94 (dd, *J* = 9.24 Hz, 3.56 Hz, 2H, H-4_ax_, H-8_ax_), 3.88 (s, 12H, 3′, 5′, 3″, 5″-OCH_3_), 3.84 (s, 6H, 4′, 4″-OCH_3_), 3.09–3.12 (m, 2H, H-1, H-5); ^13^C NMR (100 MHz, CDCl_3_): δ = 153.6 (C-3′, C-5′, C-3”, C-5”), 137.7 (C-4′, C-4″); 136.9 (C-1′, C-1″), 103.0 (C-2′, C-6′, C-2″, C-6″), 86.1 (C-2, C-6), 72.1 (C-4, C-8), 61.0 (4′, 4″-OCH_3_), 56.4 (3′, 5′, 3″, 5″-OCH_3_), 54.5 (C-1, C-5); HRESIMS *m*/*z* 447.3016 [M + H]^+^ (calcd for C_24_H_30_O_8_ 447.201344).

#### 3.4.2. Monosaccharide Composition Analysis of Polysaccharide

Five milligrams of polysaccharides was hydrolyzed with 1 mL of 2 M trifluoroacetic acid for 2 h at 90 °C. After this period, the solution was evaporated under reduced pressure and re-dissolved in 1 mL of water. An aliquot of this solution was analyzed by HPTLC, according to the method described in Section 3.3, and compared with monosaccharide standards (glucose, galactose, rhamnose, xylose, fructose, arabinose galacturonic acid, glucuronic acid).

### 3.5. HPLC-DAD Quantitative Analyses of Yangambin in RC7EE

The methanol stock solution containing 1.5 mg/mL yangambin previously purified was diluted to final concentrations of 0.3, 0.15, 0.03, 0.015, and 0.0075 mg/L. Each final solution was prepared in triplicate and analyzed (3 μL injected). The calibration curve was calculated with equal-weighted least-squares linear regression analysis of the peak area at the wavelength 271 nm against the standard nominal concentration; limit of detection (LOD) and quantitation (LOQ) were obtained as LOD = 3.3 × Sa/b and LOQ = 10 × Sa/b, respectively, where Sa and b are the estimated standard deviation and the slope of the analytical calibration function with a 95% confidence level, respectively. For yangambin, retention time was 27.7 min; calibration curve equation was y = 4×10^8^ x + 3127.1; R^2^ = 0.999; LOD = 77.9 ng; and LOQ = 236 ng.

For each oleogum sample (RC1–8), an ethanol extract (EE) was prepared as reported in Section 3.4. For each EE, three methanol stock solutions containing 3–10 mg/mL were prepared and filtered (syringe filter, hydrophilic PTFE membrane, 0.22 μm). Each final solution was analyzed (3 μL injected) by HPLC-DAD following the method described above, and yangambin amounts were quantified by the calibration curve equation.

## 4. Conclusions

In conclusion, this study reports for the first time the characterization of both the non-volatile apolar fraction and polar components of the oleogum from *C. ornifolia*, a species endemic to Socotra Island. Yangambin, a furofuran lignan interesting for its biological activities, was found for the first time in *C. ornifolia*; it has previously been reported in the *Commiphora* genus only once to date. The complex composition of the studied resin suggests the presence of other lignans, but further studies are necessary to isolate them and clarify their structures.

The HPTLC and NMR analyses of the hydrolyzed polysaccharide fraction of the *C. ornifolia* oleogum revealed the presence of arabinose, as the main monosaccharide component, followed by rhamnose, galactose, and galacturonic acid.

These first results pave the way for further phytochemical studies on the non-volatile fraction of *C. ornifolia* oleogum, which are necessary to identify other compounds that could have a pharmaceutical value, as at least partly suggested by the uses of this resin foreseen in the traditional medicine of the local population.

## Figures and Tables

**Table 1:** Yangambin content in different *C. ornifolia* samples, after extraction by ethanol maceration and evaporation under reduced pressure (see Section 3.4 for the experimental details), expressed as *w*/*w* % mean value ± standard deviation from triplicate analyses by HPLC-DAD.

*C. ornifolia* Sample	*w*/*w* % ± S.D.
RC1	6.07 ± 0.06
RC2	6.58 ± 0.42
RC3	5.84 ± 0.08
RC4	8.86 ± 0.12
RC5	4.08 ± 0.06
RC6	3.50 ± 0.02
RC7	9.05 ± 0.19
RC8	4.23 ± 0.16

**Table 2:** ^1^H NMR assignation of carbohydrates of the hydrolyzed product. The table reported the following: ^1^H chemical shift in each carbohydrate anomeric signal expressed in ppm; *J* coupling expressed in Hz; and signal assignation for α or β-CH. Rhamnose *J* coupling could not be detected (NaN) due to signal overlapping with the water signal at 4.77.

Compound	^1^H δ (ppm)	*J* (Hz)	Assignment
Arabinopiranose	5.22	3.6	α-CH
	4.50	7.8	β-CH
Arabinofuranose	5.23	3.2	α-CH
	4.51	9.3	β-CH
Galactose	5.25	3.5	α-CH
	4.57	7.9	β-CH
Galacturonic acid	5.28	4.5	α-CH
	4.58	7.9	β-CH
Rhamnose	5.10	1.7	α-CH
	4.85	NaN	β-CH

**Table 3:** ^1^H NMR carbohydrate quantification of the hydrolyzed product. Quantities for each carbohydrate are expressed as the sum of α and β anomeric signals integral ratio to the sum of α and β anomeric signals integral of rhamnose.

Monosaccharide	Ratio to Rhamnose
Total arabinose	2.3
Galactose	0.8
Galacturonic acid	0.5

## Data Availability

The original contributions presented in this study are included in the article/Supplementary Materials. Further inquiries can be directed to the corresponding author.

## Supplementary Materials

The following supporting information can be downloaded at https://www.mdpi.com/article/10.3390/plants14192999/s1: Figure S1: Sampling sites (map) and related geographical coordinates and altitudes (table) and Figure S2: ^1^H NMR spectra of the hydrolyzed product showing the anomeric signals (5.45–4.45 ppm) of carbohydrates of the hydrolyzed product.

## Abbreviations

EE	Ethanol extract
EPA	Environment Protection Authority
GC-MS	Gas chromatography–mass spectrometry
HPLC	High-performance liquid chromatography
HPLC-DAD	High-performance liquid chromatography with diode array detector
HPTLC	High-performance thin-layer chromatography
HRMS	High-resolution mass spectrometry
LOD	Limit of detection
LOQ	Limit of quantification
NMR	Nuclear magnetic resonance
PAF	Platelet-activating factor
PLC	Preparative liquid chromatography
PTFE	Polytetrafluoroethylene
RC7EE	Ethanolic extract of RC7
SPME	Solid-phase microextraction
TFA	Trifluoroacetic acid
