# Supplementary material for: Preliminary Insights into the Non-Volatile Constituents of Commiphora ornifolia (Balf.f.) J.B.Gillett Oleogum Resin from Socotra Island
Source: Plants (Basel). 2025 Sep 28;14(19):2999. doi: 10.3390/plants14192999 (PMC12526202; doi:10.3390/plants14192999)
Supplement: Supplementary file 1 [file plants-14-02999-s001.zip › plants-3874531-supplementary.pdf]

plants

Supplementary Materials

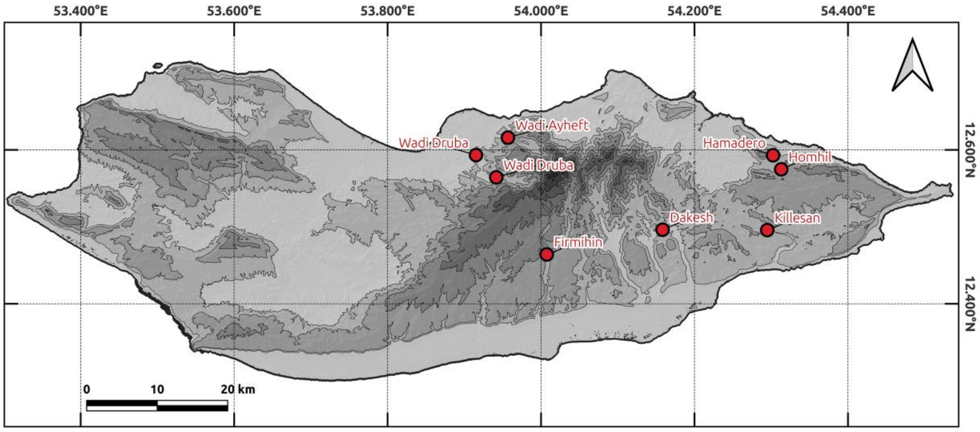

sample	identifier	locality	date	longitude	latitude	altitude
RC1	WD121122	Wadi Druba	12/11/2022	53.941675	12.564335	193
RC2	H041122	Hamadero	04/11/2022	54.302435	12.592997	568
RC3	F071122	Firmihin	07/11/2022	54.007485	12.46414	420
RC4	D151122	Dakesh	15/11/2022	54.158461	12.496286	217
RC5	H141122	Homhil	14/11/2022	54.313075	12.574818	449
RC6	WD111122	Wadi Druba	11/11/2022	53.91526	12.593262	102
RC7	K200423	Killesan	20/04/2023	54.294852	12.495767	211
RC8	WA230423	Wadi Ayheft	23/04/2023	53.956943	12.615980	110

Figure S1. Sampling sites (map) and related geographical coordinates and altitudes (table)

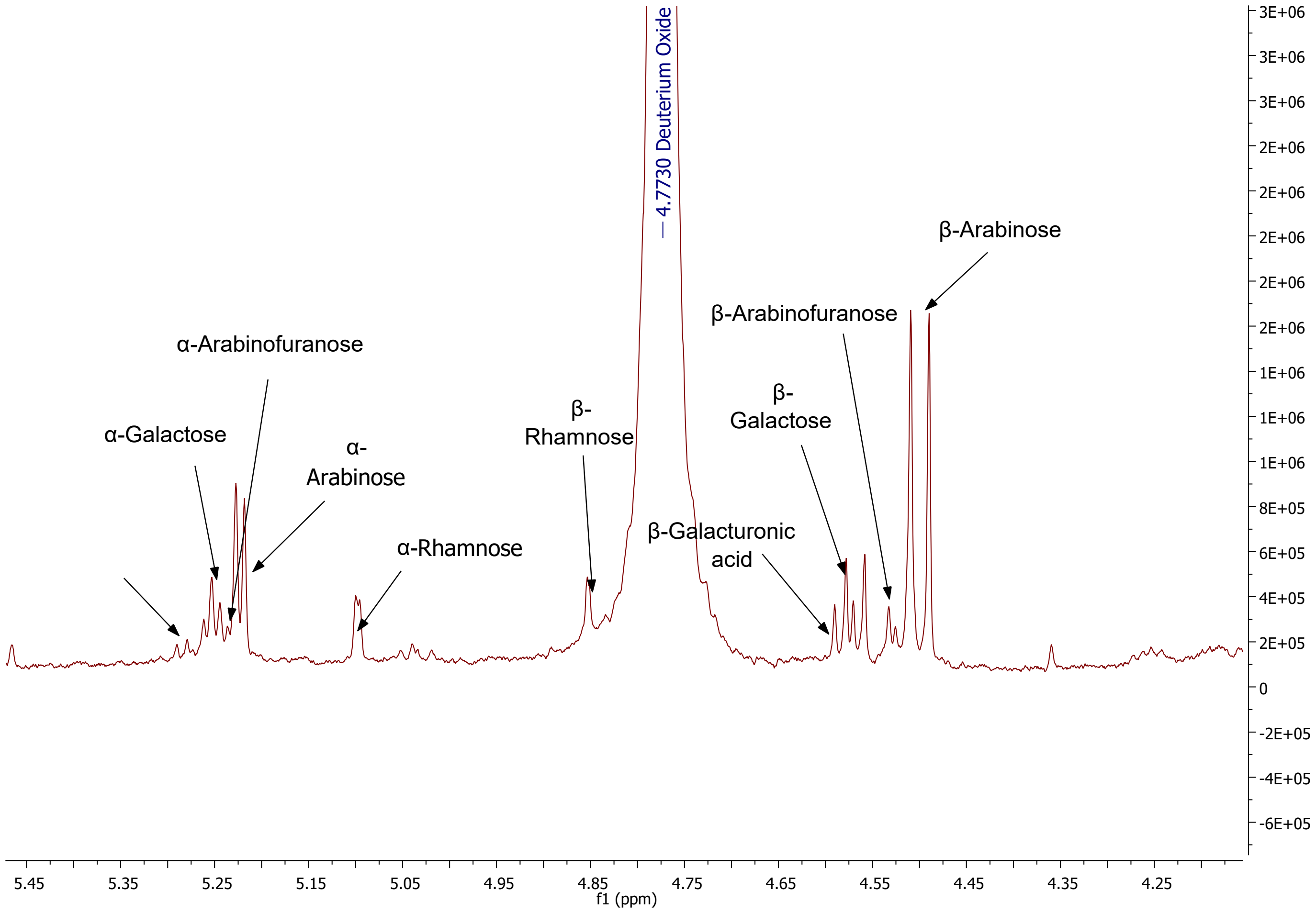

Figure S2. ^{1}H NMR spectra of the hydrolyzed product showing the anomeric signals (5.45-4.45 ppm) of carbohydrates of the hydrolyzed product.

Disclaimer/Publisher's Note: The statements, opinions and data contained in all publications are solely those of the individual author(s) and contributor(s) and not of MDPI and/or the editor(s). MDPI and/or the editor(s) disclaim responsibility for any injury to people or property resulting from any ideas, methods, instructions or products referred to in the content.
